# Developing a framework of gastronomic systems research to unravel drivers of food choice

**DOI:** 10.1016/j.ijgfs.2017.06.001

**Published:** 2017-10

**Authors:** Rosa Paula Cuevas, Annalyn de Guia, Matty Demont

**Affiliations:** International Rice Research Institute (IRRI), College, Los Baños, Laguna, Philippines

**Keywords:** Traditional rice varieties, Grain quality, Culture, Food pairings, Consumer preference

## Abstract

Nutritional and dietary interventions and the introduction of novel food products and ingredients require a thorough understanding of the drivers of food choice, which are embedded in local context and culture. We developed a framework of “gastronomic systems research” (GSR) to understand culture-specific consumer food choice, and contextualise it to a target population of urban, middle- to high-income Filipino consumers to assess the domestic niche market potential of traditional rice varieties in the Philippines. The GSR framework was contextualised through expert elicitation involving chefs and nutritionists, and validated through a consumer survey conducted during a food exposition. Using the GSR framework, we determined indicative rice consumption patterns of the target population and the specific rice quality attributes they require for specific rice-based dishes and rice consumption occasions. The GSR framework also reveals possible entry points for nutritional and dietary interventions and the introduction of novel food products and ingredients. The GSR framework, therefore, has the potential to aid policymakers and food value chain stakeholders in designing culture-sensitive and context-appropriate interventions not only to help consumers improve their diets, but also to help farmers access niche markets for novel food products and ingredients and thereby improve their livelihoods and preserve cultural heritage.

## Introduction

Little is known about how eating behaviours drive food choices (Falguera et al., 2012, French et al., 2012). Food is not just about sustenance; its consumption is embedded in rules, behaviours, and etiquette. This implies that food consumption and food choice are shaped by one׳s context, may it be cultural and/or socioeconomic. Therefore, in the quest to understand what drives people׳s food choices, we must consider a holistic approach to find knowledge gaps and intervention points in the food chain (Kittler et al., 2012), i.e., an interdisciplinary view of the complex relationships among food types and their intrinsic properties, and cultural backgrounds and societal factors of consumers. Holistic systems thinking has been used in agroecosystems and farming systems research since the late 1960s (Collinson, 2000), and since the 1990s, this approach has expanded to food value chain research (FAO, 2014). Surprisingly, spillovers of this approach to food choice research have been limited so far. Studies that took the holistic approach at identifying drivers of food choice focused more on the factors that affect the availability of healthy food to consumers (e.g., the agri-food system, the environmental system, and the health/disease system) (Hammond and Dubé, 2012) or focused on sources of variation (and their different combinations) of food choice (Jaeger et al., 2011, Loose and Jaeger, 2012, Scholderer et al., 2013). Jaeger et al. (2011) developed a framework which conceptualises individual food choice events or eating occasions as being shaped by three main factors, i.e., product, person, and place. Their descriptive approach enables observing patterns and variability in food choice events and studying factors separately or jointly. Loose and Jaeger (2012) used this framework to unravel and quantify the factors that influence beverage choices at meal times. Scholderer et al. (2013) proposed “meal mapping”, a methodology which allows segmenting meals based on empirical assessments of fit between “meal centers” and side components. Although both approaches substantially contribute to the understanding of food choice, neither of them adopts a systemic view of food choice. A hierarchical, multi-level system approach could dramatically enhance our understanding of the drivers of food choice, and facilitate the identification of multiple entry points and impact pathways for nutritional and dietary interventions and the introduction of novel food products and ingredients.

In the case of rice preferences, studies have previously been limited to determining the preferred varieties of consumers from specific regions and socio-economic classes, and the quality attributes of these varieties (e.g., Abansi et al., 1992; Calingacion et al., 2014; Champagne et al., 2010; Custodio et al., 2016; Cuevas et al., 2016; Demont et al., 2017). Studies have often associated these preferences (or a failure of accounting for them) with poor varietal adoption rates and the persistence of rice mega varieties despite the release of newer, improved varieties (Boualaphanh et al., 2011, Calingacion et al., 2014, Custodio et al., 2016, Laborte et al., 2015, Manzanilla et al., 2013). However, rarely do these studies deep-dive into the underlying factors that drive certain rice preferences.

**Fig. 1:**
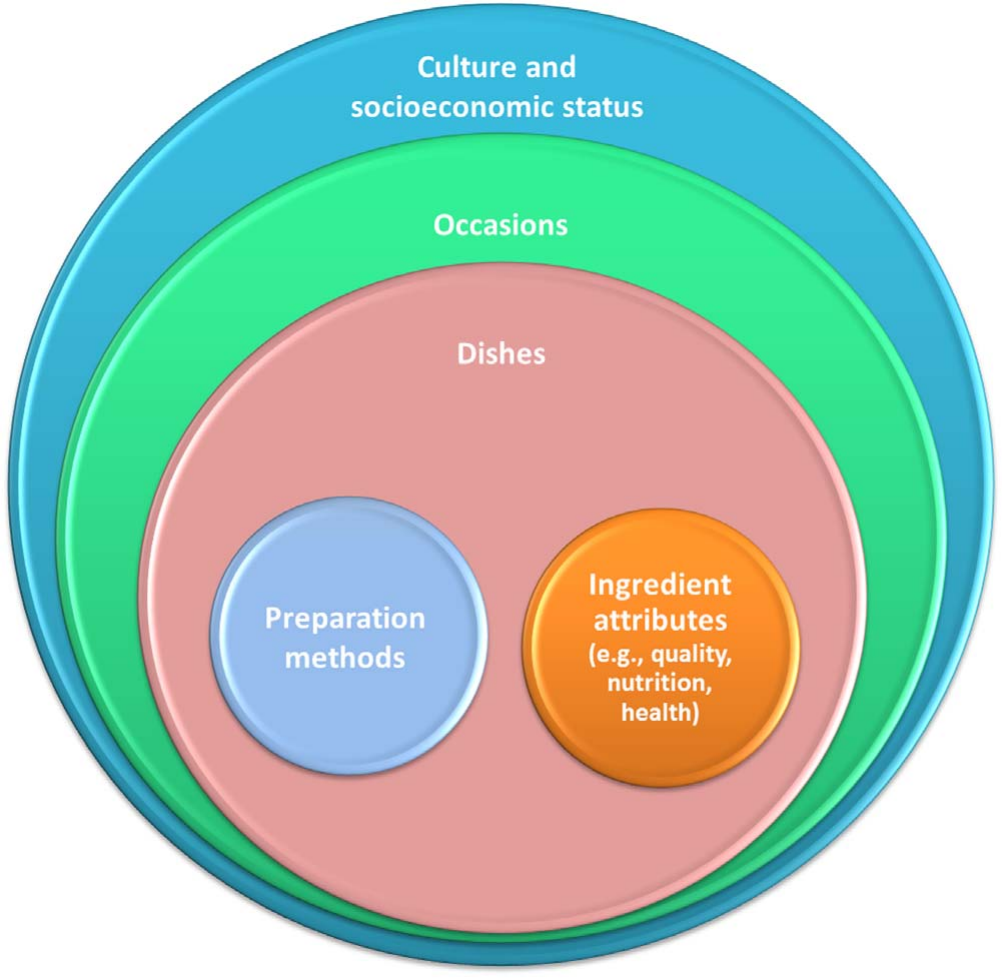
Determining consumption patterns, their drivers, and possible areas for interventions (e.g., nutritional and dietary interventions and the introduction of novel products and ingredients) requires taking a holistic view at the food system. At the consumers' end, socioeconomic and cultural contexts encompass the occasions of food consumption, the dishes paired and served during these occasions, and the derived demand for quality attributes of ingredients (such as rice) paired up in these occasions.

To address these gaps, we developed a framework for *gastronomic systems research* (GSR, Fig. 1). In the GSR framework, *gastronomy* is taken as “an appreciation and understanding of the many avenues of cooking and food production” including food quality, foodways, and culinary anthropology (Katz and Weaver, 2003, p. 102). The GSR framework embeds food choice in a context- and culture-specific system of *occasions* for food consumption (e.g., breakfast, lunch, dinner, supper, snacks, special occasions) that command certain *dishes*, which then shape derived demand for food *ingredients* carrying certain *cooking* and *eating qualities,* and *nutritional attributes*, which eventually lead to dietary and nutritional outcomes.^1^ A particular occasion determines the types of dishes, the ingredients, the cooking methods, and the mode of consumption.

**Fig. 2:**
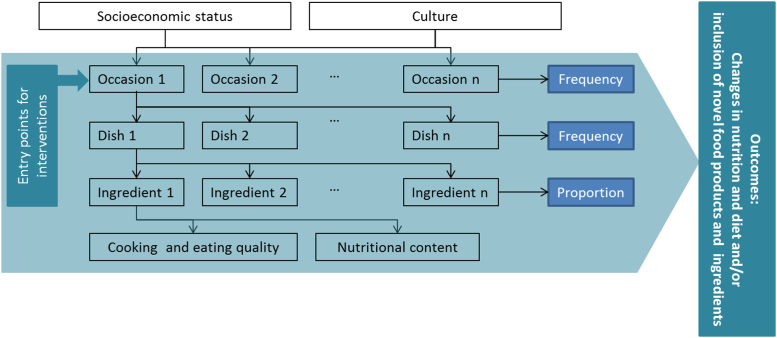
The GSR framework is a culturally sensitive and context-specific systems approach for understanding drivers of food choice and consumer behaviour and identifying entry points for nutritional and dietary interventions and the introduction of novel food products and ingredients.

The GSR framework (Fig. 2) takes cultural and socioeconomic context of the consumer as an overarching factor in explaining consumption patterns in a hierarchical framework. The hierarchical structure of the GSR framework allows identifying entry points for nutritional and dietary interventions and the introduction of novel food products and ingredients at three levels with different potentials for leverage, i.e., occasions, dishes, and ingredients. These interventions can include induced changes in food consumption patterns (e.g., nutritional and dietary interventions) and introduction of new products (e.g., novel crop varieties, food products, and ingredients).

Estimates of frequency of occurrence at the three levels enable estimating the potential reach of nutritional and dietary interventions or potential consumer demand and market shares of novel food products and ingredients. The GSR framework can also potentially provide valuable initial information for developing segmented market strategies for new food crop varieties or food products. Because of its context-specificity, one can expect that food consumption patterns at different socioeconomic strata at national and subnational levels may vary. It is projected that through the application of the GSR framework, one can potentially formulate effective, culture-sensitive, and context-appropriate interventions to improve consumer diets (e.g., nutritional and dietary interventions), and/or improve livelihoods of farmers and other actors in food value chains (e.g., identification of market niches for novel food products and ingredients).

Traditional rice varieties from the Cordillera Administrative Region (CAR) are ideal products for studying consumer food choices. These varieties have been continuously grown in terrace farms by indigenous people living in the Cordilleras, a mountainous region in the northern central part of Luzon Island, Philippines. In this region, farmers are among the poorest people (Guieb, 2016), with the incidence of poverty in the region reported at 20.6% in the first semester of 2015 (Philippine Statistics Authority, 2016). With poverty triggering mass migrations from the region, Cordilleran traditional rice varieties and traditional practices are being abandoned. Therefore, the Philippine government is driven to popularise these traditional varieties to primarily improve farmer livelihoods and food security, which can lead to the preservation of the varieties’ cultural value (and in so doing, preserve the Cordilleran terraces’ cultural heritage), and to conserve these varieties because of their capacities to withstand environmental stresses (UNESCO, 2012, IRRI, 2016, Santiaguel, 2010). These varieties are already being exported to the USA (dela Cruz, 2015, Estigoy, 2010, Fetherstone, 2010), contributing to farmers’ livelihoods, but they are currently difficult to find locally in the Philippines. For the domestic market, these Cordilleran varieties are being promoted through high-end niche markets, such as the incorporation of these varieties into recipes created by some of the Philippines’ leading chefs (de Guzman, 2016, Icamina, 2015, Lijuaco, 2016). However, it is one thing to use traditional rice varieties in fusion cuisine by avant-garde chefs; it is quite another to see this trend converted to consistent substitution of regular white rice with traditional rice varieties. Hence, in this study, the GSR framework was utilised to determine ways to improve the penetration of such traditional rice varieties in everyday rice consumption.

In this case study, we define our target market as the premium rice market and our target population as the population of urban, middle- to high-income Filipino consumers. This target segment provides important market opportunities into which rice farmers from the CAR can tap. The GSR framework was made context-specific in two independent activities: (i) an expert elicitation workshop, in which information about the ingredients, the dishes, the occasions, and their frequencies were elicited from nutritionists and chefs to determine a baseline of consumption patterns; and (ii) a consumer survey involving a rice-tasting activity.

## Materials and methods

### Contextualisation of the GSR framework

Two chefs and five nutritionists practising in the Philippines were invited to IRRI׳s headquarters in Los Baños, Laguna to participate in the expert elicitation workshop in July 2015. The chefs typically cater to urban clientele in middle to high-income classes, who, presumably, consume premium rice. The experts were tasked to contextualise the GSR framework (Fig. 2) to the rice consumption habits of a target population of urban, middle- to high-income Filipino consumers by (i) providing expert opinion on the role rice plays in food consumption patterns of the target population (i.e., identification of typical occasions of rice consumption; dishes typically eaten with rice or have rice as an ingredient per occasion; ingredients of identified dishes; cooking methods and eating techniques used for rice; level of rice and dish integration; rice grain quality required by the dishes); and (ii) identifying entry points for traditional Philippine rice varieties, and estimating probabilities of the suitability of pairing these varieties with the identified dishes for the identified occasions of rice consumption. It was assumed, when probabilities were estimated, that these traditional varieties were universally available in the market where the target consumers purchase their premium rice. This is a necessary assumption because estimating the full market potential of the product requires a focus on demand and an assumption that supply is adequate and easily accessible to the target consumers.

Experts were further asked to estimate three types of metrics. The first set of metrics is the frequencies or probabilities of occurrence of occasions, dishes, ingredients, rice preparation methods, methods for rice consumption, and suitability of traditional varieties for the identified dishes. These metrics were then used to estimate the potential (maximum attainable) market share of traditional rice varieties in total rice consumption, ${\mathrm{MS}}_{t}$, identified by the experts using Eq. (1):

(1) $${\mathrm{MS}}_{t}=\sum_{i=1}^{n}\left(\frac{F_{i}^{o}}{n}\sum_{j=1}^{k}\frac{F_{\mathrm{ij}}^{d}}{k}S_{\mathrm{ij}}\right),$$

where $F_{i}^{o}$ represents the frequency of occasion *i* per annum (*i* = 1, 2, …, *n*), *n* the total number of rice eating occasions per annum, $F_{\mathrm{ij}}^{d}$ the frequency of dish *j* during occasion *i* per annum (*j* = 1, 2, …, *k*), *k* the total number of rice-based dishes per occasion *i*, and $S_{\mathrm{ij}}$ the suitability of traditional rice varieties for dish *j* during occasion *i*.

Secondly, for each dish, we asked the experts to estimate the level of rice-viand integration on a scale from 0% to 100%. Integration of 0% means that rice and viands are totally separated in the dish and during consumption; i.e., consumers eat rice and viands separately and never pair both in their mouths. A 50% integration level means that rice and viands are somewhat separated on the dish, but are often paired during intake. In the case of 100% integration, rice and viands are completely mixed in the dish and are typically not separated during consumption. Finally, experts were asked to estimate the importance of rice quality attributes required for specific dishes, pairings with ingredients, rice-viand integration levels, and methods for rice consumption on a scale of 0–100%, 0% meaning “not important at all”, and 100% meaning “extremely important.”

### Validation of the GSR framework

To validate the contextualised GSR framework, it was important to allow target consumers to try the traditional rice varieties and allow them to perceive the eating quality attributes of the products. This experience would enable them to determine how suitable the traditional rice varieties were to dishes, and then the dishes to occasions; thus allowing for the validation of the contextualised GSR framework.

A consumer survey involving rice tasting was conducted in three one-hour sessions: one session at IRRI׳s Grain Quality and Nutrition Centre (GQNC) and two sessions during the Madrid Fusión Manila gastronomy expo (April 2015) at the SMX Convention Centre in Pasay City, Philippines. There were 76 participants, in total, in the consumer survey. The IRRI survey was participated by staff interested in trying traditional rice varieties, making them potential buyers of these varieties. The Madrid Fusión Manila survey was presumed to have participants belonging to the target consumers because the event aims to make the Philippines a gourmet destination in Southeast Asia; with internationally renowned and Michelin-starred chefs top-billing the congress that was happening alongside the expo. The expo׳s entry fees also presumably limited the access to the expo to the target consumers.

Seven traditional rice varieties were obtained as market samples: Dinorado, Tinawon White, Tinawon Red, Minaangan*,* Jekot, Ominio, and Ingudpur (Comanda, 2015, DA-BPI, 2017, Wittenberg, 2013). The labels "Tinawon White" and "Tinawon Red" refer to two rice samples whose variety names were not specified. Except for Dinorado, these traditional varieties are grown in the CAR. Since these were market samples, these samples were not subjected to further processing prior to cooking. These were washed, drained twice, and then cooked in 1:2 (v/v) ratios with water, in rice cookers.

Prior to sample presentation, participants were provided with information about the rice varieties used (e.g., where and under what conditions these were grown; the importance of preserving cultural heritage through the consumption of traditional rice varieties) through a lecture presentation. During the survey, five samples were served per participant. Drinking water was provided to allow the participants to cleanse their palates between samples. Participation in the rice tasting activity and responding to the survey questionnaire were voluntary. In the survey, participants were asked to describe the rice samples, to enumerate dishes that they think would pair well with the rice varieties, and to identify consumption occasions for these rice varieties.

In order to determine associations among occasions, dishes, ingredients, and the quality attributes of rice varieties (Fig. 2), text was extracted from the responses of the survey participants to develop a text corpus. All terms that related to consumer opinion were excluded from the descriptors (e.g., good, delicious, yummy). To reduce sparsity of terms, the non-English terms used by participants to describe the rice varieties were translated to English; similar words were combined under one keyword; and the dishes were grouped according to main ingredient and according to cooking method, following as close as possible the classification used by chefs and nutritionists in the expert elicitation workshop (Table 1) (Barretto et al., 2015, Diego, 2009); and occasions applied were “breakfast”, “lunch”, “dinner”, and “other” (special occasions such as birthdays, holidays, and parties). Text-mining, a technique that extracts patterns from unstructured text corpora (Tan, 1999), was then conducted in R (version 3.2.1) using the package “tm” (Feinerer et al., 2008). Sparsity was reduced to 22% for the descriptors and 0% for both dish type and main ingredient classifications. Correlation coefficients of the terms elicited from the survey participants were calculated in R. These coefficients indicate the degree of association among the terms and provide linkages among occasions, dishes, ingredients, cooking methods, and rice quality descriptors; thereby providing consumer-tailored information that can validate the GSR framework contextualised by expert elicitation. On the other hand, hierarchical cluster analysis was conducted in R to determine how the descriptors used by the survey participants were associated with the traditional rice varieties and how these varieties could be clustered together based on similarities in descriptions; thereby providing insights into the appropriateness of use of these traditional rice varieties in the different dishes consumed during the different occasions.

**Table 1:** The gastronomic system for rice consumption for urban Filipino premium-rice consumers, as contextualised through expert elicitation.

Context	Urban Filipinos purchasing and consuming premium-quality rice														
↓															
Occasion	Breakfast					AM/PM snack				Lunch/dinner					
Relative frequency of occasion (%)	9.6					44.8				44.8					
↓															
Dish	Silog meals			Congee/ porridge		Rice delicacies		Rice noodles		Braised meat		Risotto		Roasted chicken	
Relative frequency of dish (%)	75			25		25		75		50		10		40	
↓															
Ingredients															
Main non-rice ingredients and relative frequency of use (%)	Meat	100		Chicken	75	Sugar	100	Vegetables	50	Meat	100	Cheese	100	Meat	100
	Egg	100				Coconut milk	100	Meat	50						
						Flour	100								
Method of rice preparation	Steamed	100		Boiled	100	Boiled	50	Stir-fried	100	Steamed	100	Steamed	100	Steamed	100
	Fried	100				Steamed	50								
Level of rice-viand integration (%)	50			100		100		100		50		100		50	
Methods for rice consumption and relative frequency (%)	Spoon and fork	100		Spoon	100	By hand	75	Spoon and fork	100	By hand	50	Spoon	100	Spoon and fork	75
						Fork	25			Fork	50			By hand	25
↓															
Rice quality requirements (importance score)															
Appearance	100			50		50		0		75		100		100	
Absence of bran	80			100		100		100		75		80		90	
Aroma	70			80		50		0		75		50		50	
Taste	70			80		50		0		100		50		100	
Texture	100			75		100		50		100		100		100	

Context	Urban Filipinos purchasing and consuming premium-quality rice											
↓												
Occasion	Other special occasions						Christmas and New Year					
Relative frequency of occasion (%)	0.7						0.1					
↓												
Dish	Paella		Lechón		Rice cakes		Arroz à la valenciana		Stuffed meats		Sticky rice cakes	
Relative frequency of dish (%)	25		50		25		33		33		33	
↓												
Ingredients												
Main non-rice ingredients and relative frequency of use (%)	Meat	100	Meat	100	Sugar	100	Meat	100	Meat	100	Sugar	100
					Coconut milk	100			Vegetables	100	Coconut milk	100
					Flour	100					Flour	100
Method of rice preparation	Steamed	40	Steamed	100	Boiled	50	Steamed	40	Baked	75	Boiled	50
	Pilaf	60			Steamed	50	Pilaf	60	Boiled	25	Steamed	50
Level of rice-viand integration (%)	100		50		100		100		100		100	
Methods for rice consumption and relative frequency (%)	Spoon and fork	100	Spoon and fork	75	Fork	25	Spoon and fork	100	Spoon and fork	100	Fork	25
			By hand	25	By hand	75					By hand	75
↓												
Rice quality requirements (importance score)												
Appearance	100		100		50		100		50		50	
Absence of bran	90		90		10		90		100		100	
Aroma	30		50		50		30		50		50	
Taste	50		100		50		50		50		50	
Texture	100		100		100		100		50		100	

## Results and discussion

### Contextualisation of the GSR framework

Consumption patterns are changing in urban zones and future consumption trends must be factored in if current product development projects are to be successful. In this study, we show the applicability of the GSR framework in improving the penetration of traditional rice varieties into everyday consumption by providing information on current consumption patterns and allowing projections into future consumer preferences.

To determine the consumption patterns of Filipinos consuming premium-quality^2^ rice, the framework of the gastronomic system was contextualised to the target population, urban Filipino premium-quality rice consumers. The experts identified five typical rice-consumption occasions: (i) breakfast, (ii) morning and afternoon snacks, (iii) lunch and dinner, (iv) special occasions (e.g., birthdays, weddings, fiestas), and (v) Christmas and New Year (Table 1). It is interesting to note that most of these occasions are linked with Filipino meal times.

Based on the estimated frequencies of these occasions (Table 1, Table 2), the premium-rice consuming urban dwellers could be consuming rice around 1630 times a year. However, the calculation did not take into account possible substitutions with bread or with other staples.

**Table 2:** Potential annual market share of traditional rice varieties for different Filipino dishes and occasions.

Occasion	Estimated number of occasions annually^a^	Relative frequency of occasion per year (%)^b^	Dish	Relative frequency of dish (%)^b^	Suitability of traditional rice varieties (%)^b^	Potential annual market share of traditional rice varieties by dish (%)	Potential annual market share of traditional rice varieties by occasion (%)
Breakfast	156	9.6	Silog meals	75.0	50.0	3.6	4.8
			Congee/ Porridge	25.0	50.0	1.2	
AM & PM snacks	730	44.8	Rice delicacies	25.0	50.0	5.6	5.6
			Rice noodles	75.0	0.0	0.0	
Lunch & dinner	730	44.8	Braised meat	50.0	100.0	22.4	35.8
			Risotto	10.0	100.0	4.5	
			Roasted chicken	40.0	50.0	9.0	
Other special occasions	12	0.7	Paella	25.0	100.0	0.2	0.3
			Lechón	50.0	0.0	0.0	
			Rice cakes	25.0	50.0	0.1	
Christmas & New Year	2	0.1	Arroz a la Valenciana	33.0	100.0	0.0	0.1
			Stuffed meat dishes	33.0	100.0	0.0	
			Sticky rice cakes	33.0	50.0	0.0	
Total	1630	100.0			potential total market share:		46.6

a Estimates obtained during the expert elicitation workshop.

b From Table 1.

Thirteen dishes (two or three dishes per occasion), eaten at home or when dining out, were identified (Table 1). However, the experts felt that specific dishes might not represent the consumption patterns of Filipino urban premium-rice-consumers. They agreed that the occasion dictated the cooking method, rather than the dish itself; this is evident in the context of the Filipino culinary tradition. Filipino cuisine features several cooking methods (Alejandro, 2005, Barretto et al., 2015); such as stewing (*nilaga*), grilling (*inihaw, sinugba, inasal*), roasting (*ihurno*), steaming (*halabos, pasingaw*), sautéing (*guisado*), searing (*sankutsa*), frying (*prito*), braising (*pinalambot, pinakulob*), boiling (*pinakulo*), simmering (*inin-in, pinanukan*), and blanching (*banlian*). Different Filipino dishes may fall under the same category (Barretto et al., 2015); hence, the experts identified cooking methods if many dishes with the same methods were associated with particular rice-consumption occasions; in other cases, the experts identified specific dishes (Table 1).

For breakfast, the experts gauged that -*silog* dishes are eaten 75% of the time (Table 1). “-Silog” is a portmanteau for *sinangag* (garlic fried rice) and *itlog* (egg). The third component, the fried meat, provides the variety to this dish and is prefixed to “-silog”. These dishes are relatively easy and fast to prepare and are a means of finishing left-over food. The silog meal, therefore, fits well with the fast pace and with the high cost of urban living; indicative of the increasing opportunity cost of the food preparer׳s time and of the increasing transaction cost of the household (Reardon, 1993, Reardon et al., 2014). On the other hand, the experts estimated that congee (rice porridge) is consumed 25% of the time, perhaps because it has a longer cooking time and is most likely eaten in a more leisurely manner (i.e., weekends). Congee, which is made by boiling rice, most frequently, comes with chicken (Table 1) (Barretto et al., 2015, Besa and Dorotan, 2012). The various terms referring to congee reflecting the indigenisation of a Chinese traditional dish and its popularity in the Filipino kitchen: for example *lúgaw* (Tagalog) and *arroz caldo* (anglicised from the Spanish translation) (Kirshenblatt-Gimblett and Fernandez, 2003). Other terms indicate the variants; e.g., *goto*, congee with beef tripe (Fernandez, 2002a) and *champorado*, congee with chocolate.

The experts identified rice noodles to be the more popular snack, being consumed 75% of the time (Table 1). The Hokkien roots of the word *pancit* mean “something that is easily cooked”; in the Philippines, however, it exclusively refers to the myriad of dishes made up mainly of noodles; pancit, therefore, is another example of “cultural indigenisation" (Rodell, 2002). Pancit can be classified based on the type of noodle used to make them (Besa and Dorotan, 2012) or based on how these are cooked (Lumen, 2005). Pancit is ubiquitous in the Philippines (Kirshenblatt-Gimblett and Fernandez, 2003) and is easily accessible for the urban working class (Fernandez, 2002a). On the other hand, rice delicacies are less popular snacks, consumed 25% of the time, according to the experts (Table 1). These dishes typically involve rice (or other starchy sources), coconut, and sugar. Called *kakanin* en masse, these rice delicacies could include *palitao*, *kutsinta*, *puto*, *bibingka*, *espasol*, or *suman* (Alejandro, 2005, Besa and Dorotan, 2012). These snacks have not reached the popularity of pancit as an everyday snack for premium rice eaters in urban areas perhaps because these food items are commonly associated with churches and markets (Fernandez, 2002a); therefore, these are consumed typically over the weekend.

For lunch and dinner, the most popular dishes are braised meats, eaten 50% of the time (Table 1). Braising and stewing are similar cooking methods for tough meat cuts, except for the amount of liquid used (Barretto et al., 2015, Le Cordon Bleu International, 2011). Barretto et al. (2015) used the two terms interchangeably to describe how *adobo*, among other dishes, is cooked. Adobo has over 28,000 variations (Ocampo, 2012) and is deemed by many as the national dish in the Philippines. It is believed to be a product of Spanish and Mexican influence, although it has pre-Hispanic origins (Rodell, 2002). Other braised or stewed Filipino meat dishes illustrate Chinese, Spanish, and Malay culinary influences on Filipino cooking (Barretto et al., 2015). The second most popular dish, according to the experts, is roasted chicken (*lechón manok*), which is consumed 40% of the time (Table 1). Roasted chicken easily fits into the hectic urban lifestyle because of easy accessibility of rotisseries near residential areas. In contrast with the meat dishes, risotto, an Italian rice dish, is eaten 10% of the time, according to the experts (Table 1). The inclusion of this dish in the GSR framework for lunch and dinner suggests the increasing tendencies of urban premium-rice consumers to eat in restaurants. However, it probably has not been indigenised, as implied by its absence in Filipino cookbooks.

Special occasions tended to feature dishes with Spanish roots; indicating the relationship between the conqueror (Spain) and the conquered (the Philippines) (Kirshenblatt-Gimblett and Fernandez, 2003). Urban Filipinos, according to the experts, have retained their preferences for whole spit-roasted pig (*lechón*), which is consumed 50% of the time (Table 1). It may have Chinese and Polynesian origins but is associated with the Spanish *cochinillo asado* (Alejandro, 2005). The Filipino version of *paella* is a highly valued dish, being consumed 25% of the time (Table 1). *Arroz à la valenciana*, a dish derived from paella (Fernandez, 2002b) but which uses glutinous rice, is also associated with special occasions but more notably with Christmas and New Year (consumed 33% of the time, Table 1). Christmas and New Year are also associated with stuffed meat dishes like *relleno*. In Spain, relleno refers to the forcemeat used to stuff the chicken in *gallina rellenada* (Kirshenblatt-Gimblett and Fernandez, 2003). Filipinos indigenised the dish and developed variations, all being referred to by the partial term relleno, which now means ‘stuffed’ (Barretto et al., 2015).

Meats and meat by-products are the main ingredients in the dishes identified by the experts (e.g., consumed 90% of time for lunch and dinner, Table 1). This trend is not surprising because global trends show that increasing incomes in developing countries are one of the drivers for increasing meat consumption (Rosegrant et al., 1999).

The diversity of textures and flavours of the different dishes eaten by urban premium-rice consumers implies that the rice paired with these dishes must be diverse as well; that there are demand-driven quality attributes that influence what rice type pairs well with a certain dish. The GSR framework allows us to look deeply into these demand-driven qualities. In the Philippine context, for instance, rice is not just a basic cereal; it is the constant accompaniment that allows the nuances of Philippine dishes to shine (Fernandez, 2002b). The rice-viand pairing concept is reminiscent of food and wine pairings that are based on the complementarity of flavours between the wine and the food and the capacity of wine to enhance food flavours (Pettigrew and Charters, 2006). The estimated levels of rice-viand integration range between 50% and 100% with many dishes around 100% (Table 1), implying that rice-viand pairing is crucial in Filipino cuisine of the target population, which translates into the rice quality attributes required by consumers.

As the experts defined the type of rice that could pair well with the different dishes, they estimate that some rice characteristics are more important than others to the consumer (Table 1). *Texture* was deemed to be the most important among the experience attributes (Table 1). This is consistent with consumers’ stated preferences from surveys in the Philippines and other Southeast Asian countries (Cuevas et al., 2016, Custodio et al., 2016). Texture, in this context, is what people perceive both in their mouths and on their hands (De Groote. et al., 2014). Because of its importance, the texture of cooked rice (though primarily focused on mouthfeel) has been the subject of much study (e.g., Bett-Garber et al., 2007, Bett-Garber et al., 2013; Cagampang et al., 1973; Cameron and Wang, 2005; Champagne et al., 2001, Champagne et al., 2009; Gu et al., 2015; Juliano et al., 2012; Sitakalin and Meullenet, 2000, Sitakalin and Meullenet, 2001). Texture becomes less important, according to the experts, when rice is extruded (noodles) and when it acts as a starch source or as a thickener (in congee) (Table 1). On the other hand, the *absence of bran* is the most important search attribute for the experts (Table 1), which contradicts their inclusion of pigmented bran as a characteristic of premium rice. The bran may have effects on the processing behaviour, the texture, and the flavour of the cooked rice (Bett-Garber et al., 2013, Park et al., 2001), affecting the complementarity of unmilled rice varieties with dishes. The stark colour contrasts between coloured rice and viands (except for braised meats, which mostly have dark sauces, and for risotto, still a foreign concept) may also turn consumers off. However, unmilled rice is known to contain more nutrients and fibre than milled rice (Trinidad et al., 2013, Villareal et al., 1991), while dark pigments in the bran are associated with biomedical properties (reviewed in Rahman et al., 2016). The experts, therefore, must have included health-improving properties in defining premium rice.^2^ This suggests that despite the importance of the bran׳s absence in the *current* gastronomic system, there is scope for entry for coloured traditional rice varieties through targeted marketing interventions. And based on the importance of the bran׳s absence, the entry point with the highest potential for promoting these traditional varieties in the current gastronomic system is at lunch and dinner (Table 1, Table 2); these are the occasions in which these varieties are more easily accepted. Once these coloured rices have penetrated lunch and dinner, these will eventually be assimilated in the other rice consumption occasions. *Appearance* takes into account the “right” size and shape and the proportion of broken or damaged grains. It is an important attribute for all dishes, in various degrees, except for rice noodles (Table 1). *Taste* is less important than appearance and *aroma* scores least of all among the attributes (Table 1). This is consistent with urban consumer preferences in Southeast Asia, where taste was not mentioned in the top five most preferred rice characteristics (Custodio et al., 2016, p. 24). Perhaps the dishes that the experts had identified have strong flavours and aromas that mask the delicate flavours and aromas in cooked rice. In addition, Filipinos have a penchant to heighten the sensory experience of eating with *sawsawan* (condiments) (Barretto et al., 2015). The strong flavours and aromas of these condiments provide counterpoints to the flavours of the dishes, further masking the flavours and aromas of cooked rice.

The experts believed that Filipinos—even those consuming premium-quality rice—are not particular with the variety of the rice; instead, consumers associate sets of quality attributes to rice types and brand names, following Lancaster׳s (1966) “characteristic theory of value”. When asked to identify rice varieties that pair well with the dishes they had identified, the experts enumerated qualities of the ideal rice instead (Table 3). The exception was risotto, for which the experts identified Carnaroli as a variety best-suited for use. And just like in Table 1, the experts’ descriptions showed that texture and appearance were very important attributes (Table 3).

**Table 3:** Demand-driven quality attributes of rice based on dishes, determined by expert elicitation.^a^

Occasion	Dish	Quality attributes demanded/rice variety
Breakfast	Silog meals	
	Congee/Porridge	Moderately aromatic; softer texture; 50% waxy and 50% non-waxy
AM & PM snacks	Rice delicacies	Waxy; intermediate-amylose content
	Rice noodles	High-amylose content
Lunch & Dinner	Braised meat	Non-waxy; moderately soft
	Risotto	Bold grain; sticky; Carnaroli
	Roasted chicken	Intermediate-amylose content; long-grain rice
Other special occasions	Paella	Bold grain; waxy; high-amylose content
	Lechón	Intermediate-amylose content; long-grain rice
	Rice cakes	Waxy; intermediate-amylose content
Christmas & New Year	Arroz à la Valenciana	Bold grain; waxy; high-amylose content
	Stuffed meat dishes	
	Sticky rice cakes	Waxy; intermediate-amylose content

a The term “traditional rice varieties” here refers to those Philippine rice varieties being introduced into the domestic urban market for non-traditional use (as ingredient substitutes or paired with the dishes identified by the experts).

The experts described rice quality demanded by the different dishes based on amylose content (Table 3). It is one of the most routinely measured indicators of cooked rice quality (Custodio et al., 2016, Kumar and Khush, 1987, Tuaño et al., 2015), particularly of mechanical texture (i.e., hardness, stickiness), though reports show that the association may be weak and that other components of the rice grain also contribute to the final cooked rice texture (Bett-Garber et al., 2013, Cameron and Wang, 2005, Champagne et al., 2010, Li et al., 2016, Mestres et al., 2011). The assignment of more than one amylose content class for congee indicates that consumers mix different rice types together, either to achieve the texture demanded by the dish or to maintain the costs at a practical level (i.e., glutinous, or waxy, rice is more expensive in the Philippines). For other dishes, one׳s choice for rice depends on his/her texture preference. For instance, to prepare soft paella, the consumer may opt to use waxy rice; however, if the consumer prefers paella *al dente* with the crunchy *socarrat* (bottom layer of rice in a *paellera*), he/she may use high-amylose content rice. Rice delicacies are such a diverse class of dishes; hence waxy and intermediate-amylose rice were mentioned by the experts. Roasted and braised meats, on the other hand, are deemed to pair well with non-waxy, moderately soft rice; this is a description associated with intermediate-amylose content rice.

Table 3 also shows that the shape of the grain is an important quality attribute demanded by risotto, paella, and arroz à la valenciana (bold grains); and by roasted chicken and lechón (long grains). This specific derived demand for bold grains induced by Spanish culinary influence sharply contrasts with the trend towards long and slender rice observed in Southeast and South Asia and which is probably induced by market leaders like Jasmine and Basmati rice (Custodio et al., 2016). In contrast, the other dishes did not have such specific grain shape requirements. For example, in preparing congee, rice grains are deformed as these are boiled in excess water. On the other hand, some types of rice cakes require rice flour. Hence, for these dishes, the shape of the grains is not as important as other attributes (Table 1).

Table 2 highlights an important feature of the GSR framework: it provides product developers, marketers, and food professionals with multiple entry points for effective changes in the food system. For instance, assuming that traditional rice varieties are readily available at any time at any rice outlet, the potential market share of traditional rice varieties for consumption by urban premium-rice consumers can be estimated based on the values elicited from the experts (Table 2). Based on the experts’ estimations, traditional rice varieties can potentially attain a market share of up to 46.6% (Table 2). Lunch and dinner, and snacks are the best occasions for introducing traditional rice because these have significant shares in the frequencies (44.8% for lunch and dinner; 44.8% for AM/PM snacks, Table 2). These traditional varieties also have a place for special occasions (including Christmas and New Year); though, these occasions happen rarely in a year (0.8%, Table 2), and, hence, contribute little to the overall potential market share.

### Validation of the GSR framework in the Filipino urban premium-rice consumption context

The survey elicited four main rice consumption occasions: “breakfast”, “lunch”, “dinner”, and “other”. Similar to the experts, the consumers put “breakfast” (mentioned 121 times) as a separate category; however, “lunch” (mentioned 129 times) and “dinner” (mentioned 114 times) were kept by the consumers as separate occasions. On the other hand, it is assumed that “other” occasions (mentioned 60 times) include holidays, celebrations, and snacks that the experts identified in defining the gastronomic system framework (Table 1). Breakfast (28.5%) and lunch (30.4%) were the more popular occasions for the consumption of the traditional varieties, followed by dinner (26.9%) and other (14.2%) occasions. The popularity of breakfast and other occasions for rice consumption may seem to contradict expert-elicited information about the frequency of the two occasions (Table 1). However, the consumer data is an indicator of the traditional varieties’ appropriateness for consumption at a particular occasion, rather than an estimate of frequency of the occasion.

**Fig. 3:**
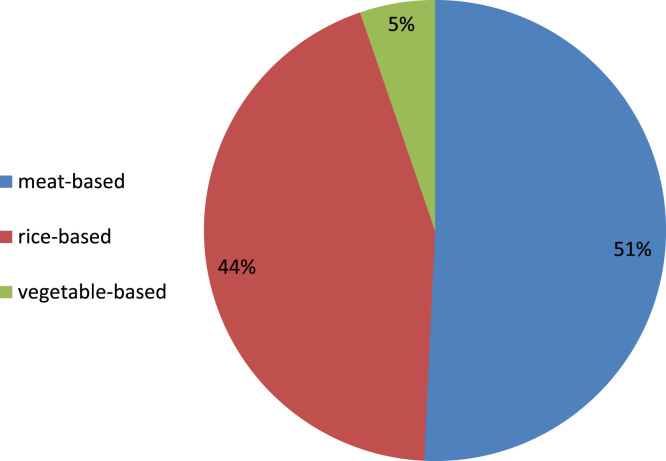
Distribution of rice-based, meat-based, and vegetable-based dishes that pair well with traditional rice varieties they described, according to the participants of the consumer survey. Meat-based dishes here pertain to anything with animal meats (poultry, fish, pork, beef).

The participants in the survey identified dishes (n = 532) suited for the occasions they had identified. These fall into three main categories based on main ingredients (Fig. 3). Half of the dishes identified (51%) had meats as main ingredients; rice is the main ingredient in 44% of the dishes; and there was a small proportion of vegetable-based dishes (5%). These indicate that the participants’ diets are composed primarily of meats, consistent with information obtained from the experts (Table 1). The dishes that the participants identified could also be grouped based on cooking method, though the number of dishes decreased when classified in this way (n = 417) because many of the participants indicated pairing with ingredients rather than with dishes.

**Fig. 4:**
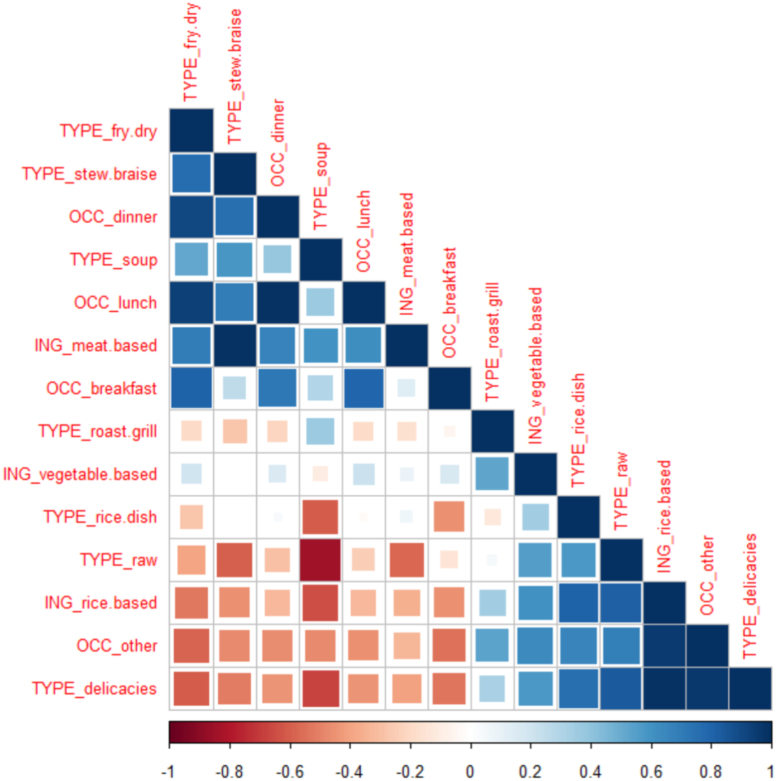
Correlation matrix for consumer-elicited rice consumption occasions (OCC) and dish types (based on cooking method, TYPE, and main ingredient, ING) based on frequencies of mention by consumers surveyed in 2015. The colours indicate the strength of the correlation; i.e., as the strength of the positive correlation increases, the blueness intensifies, while the strength of the negative correlation increases as the redness intensifies.

A correlation matrix (Fig. 4) demonstrates the links among the occasions (OCC), the dishes (classified by ING, main ingredient; or by TYPE, cooking method type), and the traditional rice varieties evaluated. The colours in the matrix indicate the strengths of the correlations: the increasing blueness corresponds with increasing positive correlation coefficients while the increasing redness indicates increasing negative correlation coefficients. High positive correlations were interpreted as good pairings while high negative correlations were associated with uncomplimentary pairings. Fig. 4 indicates that lunch and dinner are highly positively correlated, suggesting that the dishes eaten in these two occasions are similar; this is consistent with the experts’ reasoning behind grouping lunch and dinner as one occasion (Table 1). Breakfast was not as highly positively correlated with either of the two occasions, probably because the dishes consumed at breakfast are prepared using similar cooking methods as those used for dishes intended for lunch and dinner but are eaten specifically at breakfast. On the other hand, other occasions were negatively correlated with breakfast, lunch, and dinner; this implies specificity of dishes served during these other occasions.

The correlation matrix (Fig. 4) confirms that there are consumption patterns within these different occasions; i.e., the occasion dictates the dishes a consumer eats. The consumers strongly associated the three main meals of the day with fried and dried dishes; implying that Filipinos in the premium-rice market tend to consume significant proportions of fried and dried dishes in their diet. The strong association of breakfast with fried and dried food is consistent with experts’ opinions; these consumers most likely prefer fried and dried food because these are easy to prepare, just like the silog meals (Table 1). Unlike the experts, however, the consumers also associated fried and dried food with lunch and dinner. The fast-paced lifestyle of city-dwellers must be associated with this: fried and dried food can be prepared rapidly, ideal for people who carry packed lunches with them and for the busy homemaker who has to prepare a quick dinner for the family. Braises and stews, though requiring more time to prepare, have also been positively correlated with lunch and dinner, though less intensely than fried and dried food. The association of braises and stews with lunch reflects, possibly, consumers’ dining-out patterns for this occasion. For instance, people who live too far from their offices most likely eat in restaurants because there is no time to pack their lunches in the morning. This makes braised and stewed viands viable options for lunch. Also because of the long preparation time, braises and stews are probably eaten more at home, at dinner, as indicated by the slightly stronger positive correlation (Fig. 4). Roasted and grilled viands, in contrast, were not strongly correlated with the three regular daily meals. Instead, these are moderately associated with other occasions. Filipino street food tradition features barbecued meats; however, these are also associated with consumers belonging to lower income classes (Fernandez, 2002a), so perhaps, these grilled meats play a minor role in the premium-rice consumers’ diets. For premium-rice consumers, the moderate association may probably be linked with the consumption of lechón, deemed a status symbol during special occasions, and with roasted chicken; both of these dishes are included in the GSR framework (Table 1). Delicacies, on the other hand, were strongly and positively correlated with other occasions; a similar concept as that elicited from the experts. Delicacies were not complementary to the three regular rice consumption occasions, indicating that these dishes are eaten separately from these meals. These delicacies, it seems, are considered as snacks.

Thus, the consumer survey seems to confirm that the occasion dictates the dishes one eats. And since Filipinos eat rice on all these occasions and rice quality is highly diverse, some rice varieties must be more appropriately paired with the dishes consumed in these different occasions. There were 103 unique terms used by the participants in the consumer survey to describe the seven traditional rice varieties evaluated. The list was shortened to 41 terms through sparsity reduction. The top two most frequently used words to describe the rice varieties evaluated were *soft* and *sticky* (Supplementary Fig. 1), suggesting that the participants in the survey viewed texture to be a major criterion in defining the quality of cooked rice. The results agree with the experts’ estimations (Table 1), particularly with their emphasis on defining the appropriate rice types based on a measure of texture, i.e., amylose content class (Table 3). This observation also agrees with previous reports stating that texture is the main attribute of cooked rice, according to consumers (reviewed in Mestres et al., 2011). For the participants in the consumer survey, texture descriptors (frequency = 570) ranked first in frequency followed by taste descriptors (frequency = 350) and then by colour descriptors (frequency = 102). This is, again, consistent with other consumer surveys in the Philippines and Southeast Asia (Cuevas et al., 2016, Custodio et al., 2016).

**Fig. 5:**
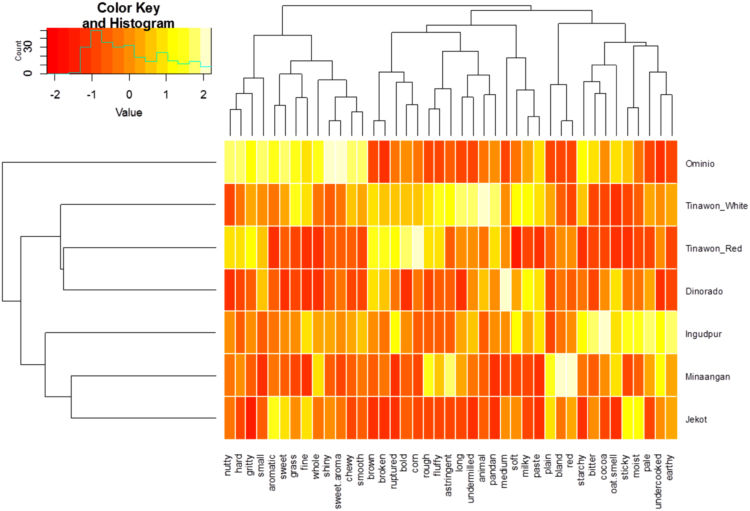
Cluster analysis of the descriptors of the seven rice varieties evaluated during the consumer survey conducted in 2015. The colours in the heat map correspond to the strength of the association between the descriptor and the variety; i.e., the weakest variety-descriptor associations are in the reds while the strongest associations are in the yellows.

The comparison of frequencies of mention of the different descriptors for the seven varieties is presented as a heatmap in Fig. 5 such that high frequencies are yellows while low frequencies are reds. The seven traditional rice varieties can be grouped into three main groups based on how often the descriptors were used on these varieties: (1) Ominio; (2) Tinawon White, Tinawon Red, and Dinorado; and (3) Ingudpur, Minaangan, and Jekot. Ominio was in its own cluster because of its unique profile. Among the varieties, it was matched most frequently with descriptors such as nutty, sweet, grassy, and sweet aromatic. In contrast, Ingudpur, Minaangan, and Jekot were associated with words such as cocoa, starchy, oat, and earthy; while Tinawon White, Tinawon Red, and Dinorado were associated with words such as corn, animal-like, pandan, and milky. Appearance-wise, Ominio was distinct from Tinawon White, Tinawon Red, and Dinorado because Ominio had small and whole grains while the three varieties were broken, ruptured and had long, medium, and bold grains. Jekot, Minaangan, and Ingudpur were mainly red varieties while the tinawons and Dinorado were mainly described as brown or undermilled. Ominio was described primarily as hard, smooth, and chewy while the tinawons and Dinorado were described as rough, fluffy, and soft; the red varieties were mainly sticky and moist. All these differences in descriptions suggest that the different varieties will pair well with different dishes. Varieties in the same group may probably be good substitutes for each other, much better than substitutions using varieties in other clusters.

**Fig. 6:**
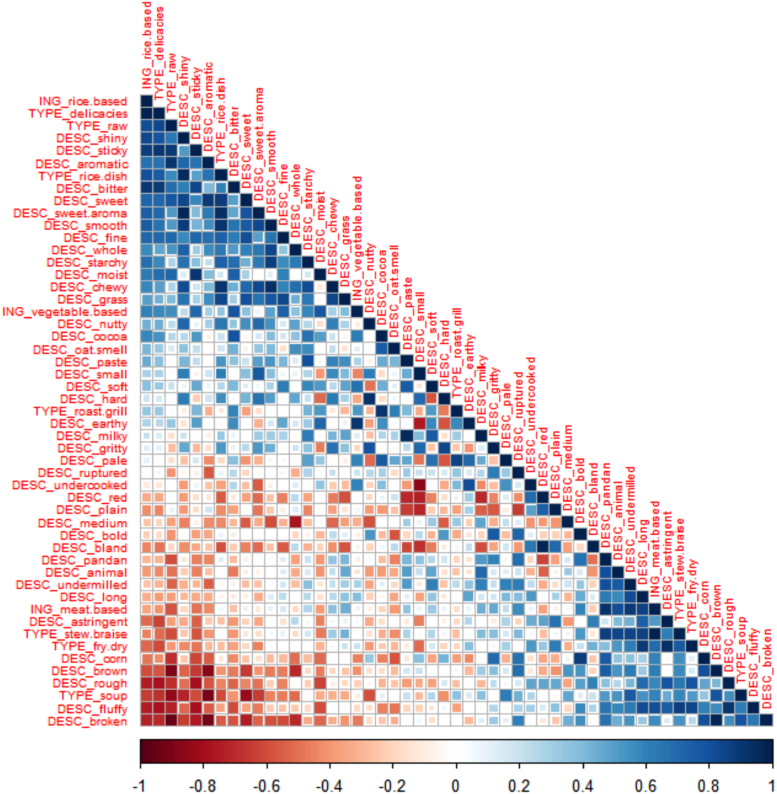
Correlation matrix for dish types (based on cooking method, TYPE, and main ingredient, ING) and rice descriptions (DESC) based on frequencies of mention from consumers surveyed in 2015. The colours indicate the strength of the correlation; i.e., the strength of the positive correlation increases as the blueness intensifies while the strength of the negative correlation increases as the redness intensifies.

The correlations among these descriptors and the dishes elicited from the consumers were then calculated to determine if rice quality descriptors tend to pair with certain dishes. Results (Fig. 6) showed that meat-based dishes were projected to go well with undermilled, fluffy, long-grain rice with pandan aromatics and animal-like odours (the tinawons and Dinorado). The rice varieties with shiny and sticky grains and with bitter taste were deemed best for rice-based dishes (Ominio and the red rices). Vegetable-based dishes were expected to be complemented by rice with earthy flavours (the red rices). Meanwhile, delicacies tended to be associated with rice that are sticky and bitter (the red rices) while fried and dried dishes were paired with long-grain, fluffy rice that have rough surfaces and astringent flavour (the tinawons and Dinorado). Raw dishes were thought to work best with aromatic rice (Ominio); broken and brown grains were strongly negatively associated with raw dishes. For rice-based dishes, the consumers projected that shiny, smooth, and chewy rice with sweet taste and aroma, and a grassy flavour, work best (Ominio). Grilled and roasted dishes were paired with pale rice grains having flavours associated with cocoa (the red rices). Soupy dishes were associated with broken grains that are not aromatic and are not sweet (the tinawons and Dinorado). Finally, stewed and braised dishes were thought to work well with long-grain fluffy rice having pandan- and animal-like odours (the tinawons and Dinorado).

The consumers’ evaluations of rice varieties and their identified rice-dish pairings (Fig. 6) were quite different from those of the experts (Table 3). However, there were commonalities: (1) roasted meats tended to be paired with long-grain fluffy rice; (2) rice-based dishes were paired with sticky rice; and (3) sticky rice also worked well with delicacies. This illustrates the importance of validation in developing the GSR framework: by actually evaluating the product, target consumers can provide information that can be used to refine the GSR framework, leading to a better understanding of the system.

**Fig. 7:**
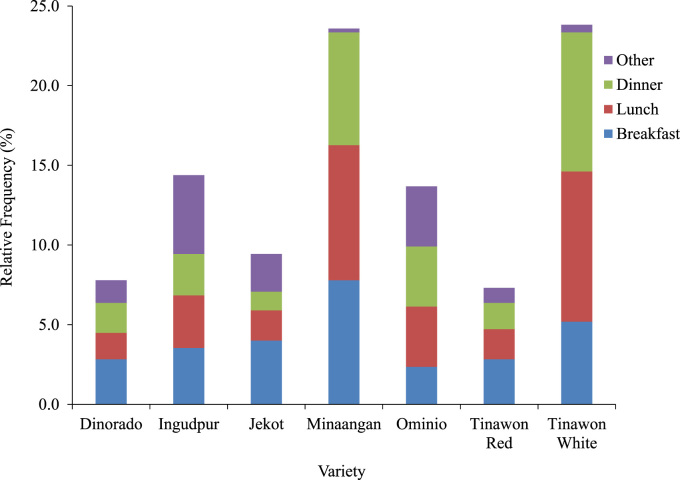
Relative frequency of projected use of the traditional varieties by occasion, according to consumers surveyed in 2015.

The different rice-dish pairings and the different occasions in which the consumers see themselves eating these traditional rice varieties suggest that these varieties have different consumer-use patterns. Fig. 7 shows the relative frequency of consumers’ projected use of the different traditional varieties according to occasion. Minaangan and Tinawon White are seen to be the most popular among the seven varieties based on mentions of projected use by occasion. Most of the projected uses for these two varieties are in breakfast, lunch, and dinner. Minaangan was seen to be of more use at breakfast than Tinawon White, which was deemed to be more appropriate for lunch and dinner. In contrast, the other four traditional varieties had fewer matches to the rice-consumption occasions and these were seen as varieties the consumers would consume during other occasions. Ingudpur, in particular, was evaluated to be more appropriate during other occasions than during breakfast, lunch, or dinner. Ominio, on the other hand, was considered to be equally appropriate for lunch, dinner, and other occasions; it was mentioned less frequently for breakfast. Jekot and Tinawon Red were even less frequently matched to the occasions identified by the consumers; however, the relative frequencies indicate that these two traditional varieties were best matched, according to consumers, to breakfast than to the other three occasions. It was interesting to note that the projected frequency of use for Dinorado was very similar to that of Tinawon Red.

These projected consumption patterns (Fig. 7) indicate that there are multiple entry points for these traditional varieties into the current gastronomic system of rice consumption of urban premium-rice consumers in the Philippines. Because Minaangan and Tinawon White have the most matches with occasions, these two varieties have the highest potentials of entering this target market for daily consumption. On the other hand, Ingudpur and Ominio have the most potential to penetrate the market for other occasions. Due to similarities in frequencies of occasion matches, Tinawon Red and Jekot seem to have the biggest potential to substitute for Dinorado, particularly since Tinawon Red and Dinorado were grouped together (Fig. 5). Such indications can aid in the development of context-specific and culturally sensitive marketing strategies for such rice varieties. If these strategies are successful, these can aid in promoting the conservation of cultural heritage through consumption of traditional rice varieties by urban consumers.

## Conclusion

In this paper, we developed a framework for gastronomic systems research (GSR) which can be used to unravel drivers of food choice and identify multiple entry points for nutritional and dietary interventions and the introduction of novel food products and ingredients. We contextualised the GSR framework and validated it with a target population of urban, middle- to high-income Filipino consumers to assess the domestic niche market potential of traditional rice varieties in the Philippines. The case study clearly demonstrated that Philippine gastronomy follows a culture- and a context-specific system: the occasion dictates the type of dishes that are being served, which then dictate the type of rice that people pair with the dishes. Through the GSR framework, we were able to establish, specific for a target population of urban premium-rice consumers from middle to high-income classes, a baseline of occasions, dishes, and ingredients that dictate demand for certain quality attributes from the rice varieties that they pair best with. Through estimations of frequencies of occasions and dishes, the GSR framework provides further insights on entry points for traditional rice varieties from the CAR, Philippines. By further combining this information with estimations of suitability, experts estimated the potential (maximum attainable) market share of traditional rice varieties to be 46.6%. In order to realize this potential, value chain actors will have to apply an optimal marketing mix to capture and promote traditional rice products’ unique selling points and differentiate them from other products in the premium rice market (e.g., Jasmine, Japonica, brown rice, Basmati). The optimal marketing mix consists of the “four Ps”: (i) a product with intrinsic (texture, aroma, taste, etc.) and extrinsic (branding, packaging) attributes tailored to consumer preferences; (ii) a pricing strategy based on positioning of traditional rice in the premium market and price elasticity considerations; (iii) a promotion strategy (e.g., advertising, social media marketing, video marketing, etc.) to disseminate relevant information to consumers about traditional rice and differentiate it from the other premium rice products (e.g., through its distinct cultural heritage); and (iv) the place where the product will be sold (e.g., in supermarkets, food fairs, fair-trade shops, domestic markets, international markets, etc.). This paper demonstrates the value of the GSR framework as a potentially powerful tool for increasing market share for traditional rice varieties and thereby expanding market opportunities of resource-poor farmers and preserving their cultural heritage.

At a broader scale, policy makers and product developers can apply the GSR framework to identify multiple entry points for context-specific and culturally acceptable nutritional and dietary interventions and the introduction of novel food products and ingredients. For example, the GSR framework can be used to devise promotional tools for the consumption of healthy ingredients (e.g., vegetables) or for enhancement of ingredient diversity through novel dishes, or for occasions (e.g., a healthy snack every morning). Distributors can apply the GSR framework to promote novel products by targeting the occasion (e.g., breakfast cereals) and/or a novel dish based on the optimal pairing of the novel product (e.g., traditional rice) with certain ingredients. Therefore, embedding food choice in a GSR framework can make nutritional and dietary interventions and the introduction of novel food products and ingredients more effective, which may expand opportunities for resource-poor farmers to tap into growing urban markets and thereby improve their livelihoods and preserve cultural heritage.

## Funding sources

This study was supported by the CGIAR Research Program on Rice, and the Bureau of Agricultural Research of the Department of Agriculture of the Philippines. This research has also been funded by the Drivers of Food Choice (DFC) Competitive Grants Program (Grant no. OPP1110043), which is funded by the UK Government׳s Department for International Development and the Bill & Melinda Gates Foundation, and managed by the University of South Carolina, Arnold School of Public Health, USA; however, the views expressed do not necessarily reflect the UK Government׳s official policies.

## Appendix A Supplementary material

Supplementary material Word cloud of descriptors used to describe the seven rice varieties evaluated during the consumer survey in 2015. In this word cloud, the font size of each word indicates the relative frequency of mention and the two most frequently used words are in blue font.

## Footnotes

1 One can easily draw a parallel with farming systems research. There, crop choice has long been recognised to be driven by, and embedded in, a hierarchical structure of a cultural, historical, political, socioeconomic, market, and biophysical environment. This environment then shapes farming systems, which, in turn, combine certain cropping systems, which command certain agricultural practices, and which eventually lead to a certain economic, social, and environmental outcome (Collinson, 2000).

2 Premium rice was defined by the experts as rice with “superior” properties, including aroma and pigmented bran. This definition implies an emerging shift in consumer preferences because traditionally, white rice is preferred for table rice (de Leon, 2005).
