# Supplementary material for: Developing a framework of gastronomic systems research to unravel drivers of food choice
Source: Int J Gastron Food Sci. 2017 Oct;9:88–99. doi: 10.1016/j.ijgfs.2017.06.001 (PMC5632960; doi:10.1016/j.ijgfs.2017.06.001)
Supplement: Supplementary file 1 — Supplementary material Word cloud of descriptors used to describe the seven rice varieties evaluated during the consumer survey in 2015. In this word cloud, the font size of each word indicates the relative frequency of mention and the two most frequently used words are in blue font. [file mmc1.doc]

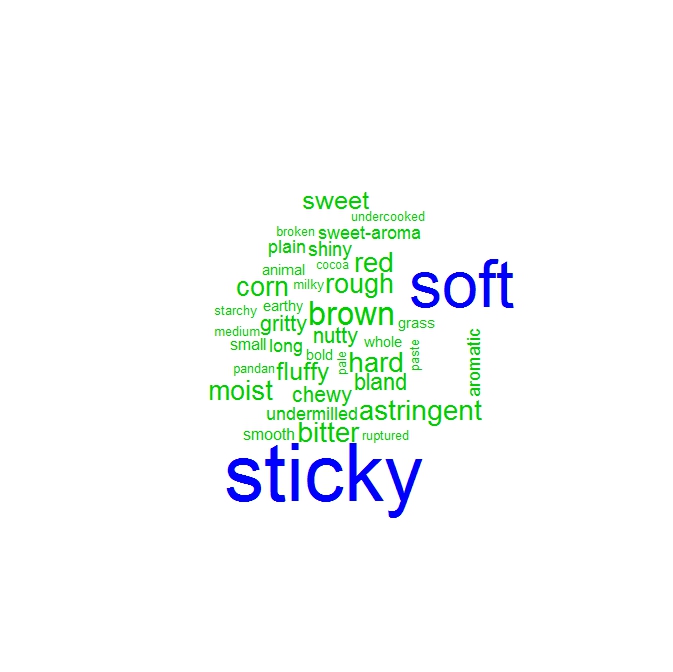


Supplementary Fig. 1. Word cloud of descriptors used to describe the seven rice varieties evaluated during the consumer survey in 2015. In this word cloud, the font size of each word indicates the relative frequency of mention and the two most frequently used words are in blue font.
